# Supplementary material for: Miocene Tropical Forests in South China Shaped by Combined Asian Monsoons
Source: Plants (Basel). 2025 Nov 25;14(23):3599. doi: 10.3390/plants14233599 (PMC12694115; doi:10.3390/plants14233599)
Supplement: Supplementary file 1 [file plants-14-03599-s001.zip › Figure S1.docx]

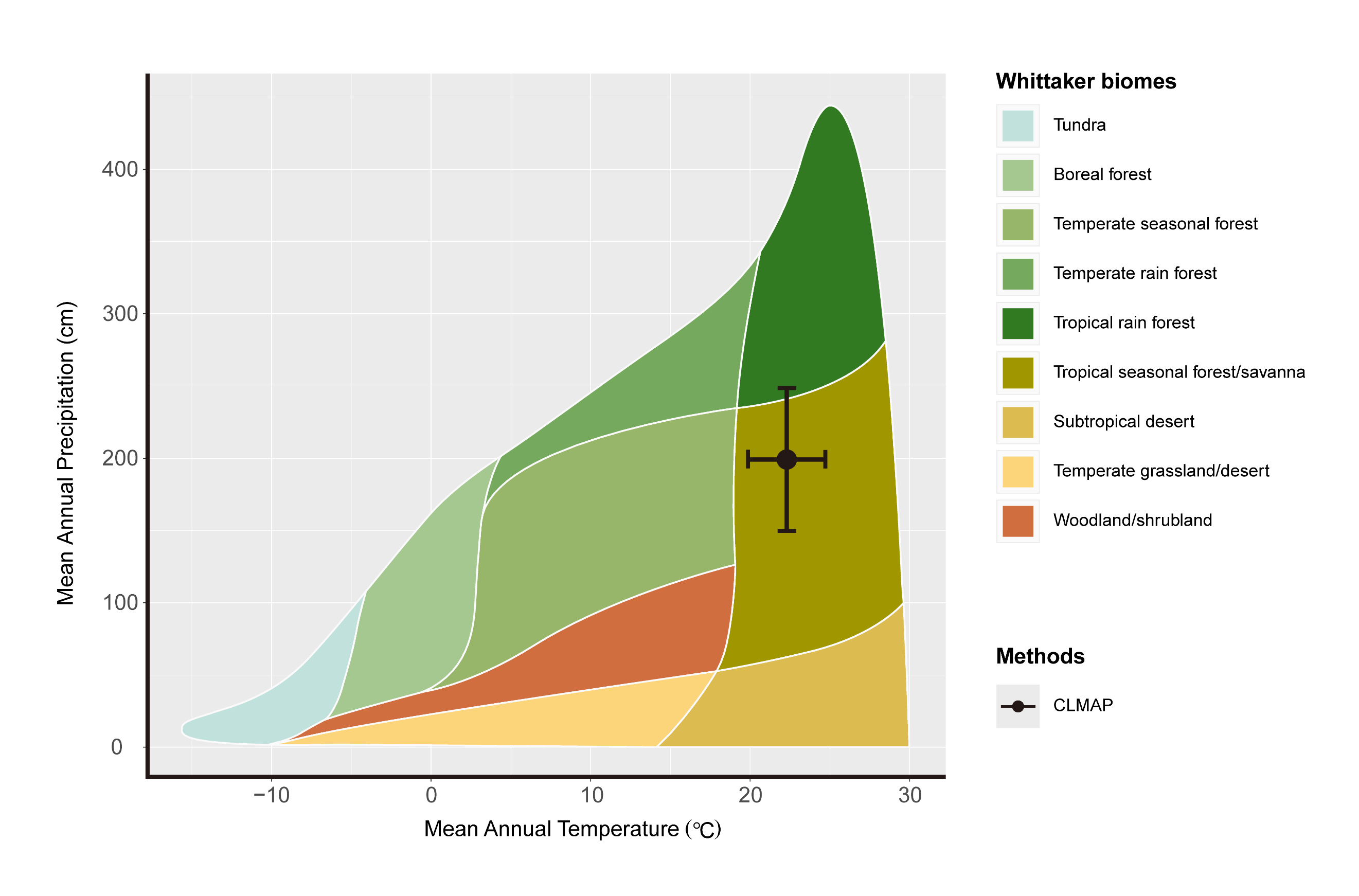

Figure S1. Whittaker Biome diagram showing the Miocene climatic position of the Guiping Basin.

The Miocene climatic position of the Guiping Basin is shown on a Whittaker biome diagram generated with the plotbiomes dataset (Whittaker boundaries, digitized by Ștefan and Levin [58] and distributed with the plotbiomes R package). Mean annual temperature (MAT) and mean annual precipitation (MAP) estimates for Guiping—22.3 ± 2.27°C and 199.1 ± 48.1 cm (Table 1), respectively—are overlain on the diagram. The Guiping Miocene point falls on the boundary between tropical seasonal forest/savanna and tropical rain forest, lying closer to the tropical seasonal forest zone than to the core tropical rain forest zone.
